# Overexpression of septin-7 inhibits melatonin-induced cell apoptosis in human fetal osteoblastic cells via suppression of endoplasmic reticulum stress

**DOI:** 10.3892/mmr.2021.12538

**Published:** 2021-11-22

**Authors:** Xiaotong Meng, Yue Zhu, Lin Tao, Sichao Zhao, Shui Qiu

Mol Med Rep 17: 4817-4822, 2018; DOI: 10.3892/mmr.2018.8449

Subsequently to the publication of this paper, an interested reader drew to the authors’ attention that Figs. 2 and 4, featured on p. 4820 and 4821 respectively, contained apparently matching control β-actin western blots.

The authors have consulted their original data, and realized that the control western blot images were inadvertently selected incorrectly for Fig. 2. The corrected version of Fig. 2, showing the relevant β-actin bands for Fig. 2, is shown on the next page. Note that the errors in Fig. 2 did not significantly affect the results or the conclusions reported in this paper, and all the authors agree to this Corrigendum. The authors are grateful to the Editor of *Molecular Medicine Reports* for allowing them the opportunity to publish this corrigendum, and apologize to the readership for any inconvenience caused.

## Figures and Tables

**Figure 2.**
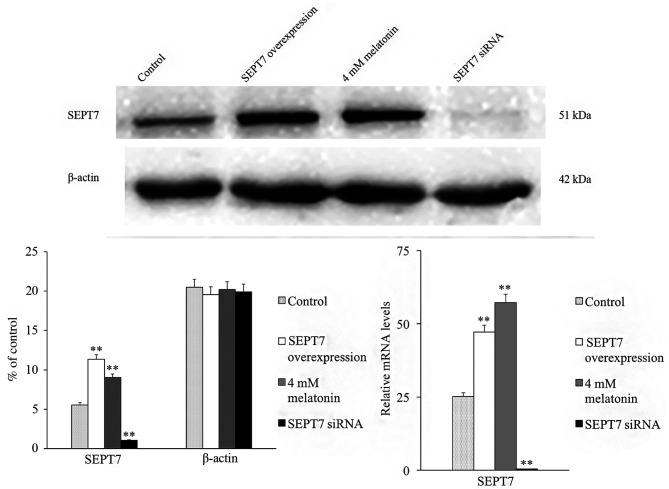
Protein and mRNA expression of SEPT7 following treatment with SEPT7 overexpression plasmid or SEPT7 siRNA in human osteoblastic cells line hFOB 1.19. Each bar represents the mean ± standard error of the mean of three independent experiments. *P<0.05 and **P<0.01 vs. control cells. SEPT7, septin-7; si, small interfering.

